# Inequalities in caries among pre-school Italian children with different background

**DOI:** 10.1186/s12887-022-03470-4

**Published:** 2022-07-23

**Authors:** Guglielmo Campus, Fabio Cocco, Laura Strohmenger, Thomas Gerhard Wolf, Araxi Balian, Antonella Arghittu, Maria Grazia Cagetti

**Affiliations:** 1 Department of Restorative, Preventive and Pediatric Dentistry, University of Bern, Freiburgstrasse 7, 3012 Bern, Switzerland; 2 Department of Surgery, Microsurgery and Medicine Sciences, School of Dentistry, University of Sassari, Viale San Pietro 3/c, 07100 Sassari, Italy; 3 School of Dentistry, Sechenov University, 119991 Moscow, Russia; 4 Department of Biomedical, Surgical and Dental Sciences, University of Milan, Via Beldiletto 1, 20142 Milan, Italy; 5 Department of Periodontology and Operative Dentistry, University Medical Center of the Johannes Gutenberg-University Mainz, 55131 Mainz, Germany; 6 Direzione Igiene E Controllo Delle Infezioni Ospedaliere, University Hospital of Sassari, Via Padre Manzella 4, 07100 Sassari, Italy

**Keywords:** Caries, Health care disparities, Inequalities, Social determinants, Children, Pediatric dentistry

## Abstract

**Background:**

The study was aimed to describe caries prevalence and severity and health inequalities among Italian preschool children with European and non-European background and to explore the potential presence of a social gradient.

**Methods:**

The ICDAS (International Caries Detection and Assessment System) was recorded at school on 6,825 children (52.8% females). Caries frequency and severity was expressed as a proportion, recording the most severe ICDAS score observed. Socioeconomic status (SES) was estimated by mean a standardized self-submitted questionnaire filled-in by parents. The Slope Index of Inequality (SII) based on regression of the mid-point value of caries experiences score for each SES group was calculated and a social gradient was generated, children were stratified into four social gradient levels based on the number of worst options. Multivariate regression models (Zero-Inflated Negative Binomial logistic and logistic regression) were used to elucidate the associations between all explanatory variables and caries prevalence.

**Results:**

Overall, 54.4% (95%CI 46.7–58.3%) of the children were caries-free; caries prevalence was statistically significant higher in children with non-European background compared to European children (72.6% *vs* 41.6% *p* < 0.01) and to the area of living (*p* = 0.03). A statistically significant trend was observed for ICDAS 5/6 score and the worst social/behavioral level (Z = 5.24, *p* < 0.01). Children in the highest household income group had lower levels of caries. In multivariate analysis, Immigrant status, the highest parents’ occupational and educational level, only one kid in the family, living in the North-Western Italian area and a high household income, were statistically significant associated (*p* = 0.01) to caries prevalence. The social gradient was statistically significant associated (*p* < 0.01) to the different caries levels and experience in children with European background.

**Conclusions:**

Data show how caries in preschool children is an unsolved public health problem especially in those with a non-European background.

**Supplementary Information:**

The online version contains supplementary material available at 10.1186/s12887-022-03470-4.

## Introduction

The WHO resolution on oral health adopted on 27^th^ May 2021 [1] underlines the need to improve oral health worldwide. In many developed and developing countries oral health care is not covered in primary health care, where high out-of-pocket expenditures for oral health services and care particularly affect disadvantaged populations.

Ending caries and ensuring good oral health is vital for general health at the very early years of children and the quality of life both of children and parents [2]. However, strengthening health-promoting environments in key settings will require multisector action and Health in All Policy approaches [1]. An unequal skewed distribution of caries figures is reported in several countries [3], where differences in disease figure might be explained with the different access to public Oral Health Services combined to different behaviors such as sugar intake, fluoride use and oral hygiene habits.

Children from low-income households have shown a higher caries prevalence than those from high-income households, as well as a greater occurrence of untreated dental caries [4]. Socio-Economic status (SES) is measured by a series of indicators. Considering the socioeconomic status of children, the income of the family, the occupational status and the educational level of parents are among the most important SES indicators.

Measures of socioeconomic inequality in health are a useful tool to assess the scale of inequalities between population groups and within populations [5, 6]. The indices are sensitive to the ordering of social groups and their health gradients, meaning that a negative score indicates an increment of the disease outcome as socioeconomic disadvantage increases [5]. These gradients in health runs from the top to the bottom of the socioeconomic range suggesting the existence of a social gradient [7].

Also, ethnicity and immigrant status, have been found to be significantly related to the presence of caries in the primary dentition [8, 9] without differences among countries from which the parents had immigrated from [10]. Furthermore, children whose families were in the lowest household income quintile, regardless of immigrant group, had the highest caries experience. Parents of children with immigrant background often report low frequency of daily toothbrushing, high consumption of sugar containing foods and infrequent dentist check-ups [11, 12].

Migration is a growing occurrence around the world as well as in Italy. In 2017, the immigration flow amounted to over 343 thousand people with a significative increase over the previous year (+ 14%) [13].

In Italy, the Public Health System has suffered over the last two decades a continuous reduction of the fraction of Gross National Product spent on healthcare; oral healthcare is almost completely provided by private practitioners and mainly financed by patients’ direct payment. In this situation, the spending possibility of the family plays an essential role on the access to dental care.

In 2016, an epidemiological survey called “National pathfinder on children’s oral health in Italy” was promoted by the WHO Collaboration Centre for Epidemiology and Community Dentistry of Milan. It was the second National Survey conducted in Italy on children’s oral health. The project aimed to examine three groups of children at the age of 4, 6 and 12 years old; the examinations started in November 2016 and were completed in June 2017.

The present study is aimed to:

i) describe caries prevalence and severity and socioeconomic inequalities among Italian preschool children with European and non-European background;
ii) compare these inequalities using both absolute and relative measures of socioeconomic inequality in health and.
iii) to explore the potential presence of a social gradient in dental health.

 

## Materials and methods

### Study design and study population

The study was designed as a cross sectional survey conform to the STROBE guidelines; data were obtained from the second Italian Pathfinder on oral health, a population-based cross-sectional survey of Italian children aged from 4 to 12 years [4]. The survey protocol was approved by the ethical committee of the University of Sassari (Italy) (AOUNIS: 29/16). In 2017 the Italian population was of 60,589,445 (29,445,741 males and 31,143,704 females); 1.62% were of 3–4 years old.

Sample procedure and data collection were previously described [4].

A multistage cluster sampling was performed as previously reported [14], considering the Italian sections as strata: North-West, North-East, Central, South and Insular Italy. In the second stage the counties of the sections and then the schools (kindergartens) were chosen at cluster level with proportional random selection of participants. A sample size for each stratum was calculated based on an assumed prevalence of dental caries [14] with a standard error of 0.05 and a design effect of 2.5 [15]. The number of 5,100 Italian children aged 4 years was increased by 10%. This strategy provided a sample that was self-weighting. In total, 7,051 children were recruited and 6,825 were examined; 173 children with no parental consent and 53 not present in the classroom at the moment of the examination were excluded. The study sample represented 1.16% of the total Italian population aged 3–4 years attending kindergartens (585,985 children with a frequency of 59.7%.) [13].

Subjects were examined at school by calibrated examiners [16], using a plain mirror (Hahnenkratt, Königsbach, Germany) and the WHO ballpoint probe (Asa-Dental, Milan, Italy) under artificial light. Caries data was recorded using the two-digit codes related to ICDAS for each tooth surface.

### Clinical outcomes

Two units of analysis were selected: subject and tooth. The frequency of caries was the outcome variable analyzed by subject, while the most severe ICDAS score observed was the variable used at tooth level. Each tooth was recorded as caries-free (ICDAS 0), enamel lesion (ICDAS 1/2), pre-cavitated lesion (ICDAS 3/4), cavitated lesion (ICDAS 5/6), filled teeth due to caries (Ft) and missing teeth due to caries (Mt). At subject level, the sum of teeth caries affected (ICDAS ≠ 0), filled or extracted due to caries was calculated as Caries Experience (CariesEx).

### Socioeconomic status/explanatory variables

European children were defined as children whose both parents were born in one of the European countries, while non-European children were coded as those whose at least one parents born outside Europe. Children were stratified based on their Socioeconomic status and behavioral habits of children/parents/caregivers (prolonged breastfeeding, the use of pacifier at night, brushing frequency, the frequency of a cariogenic diet and smoking habit of the parents) were estimated by mean a standardized self-submitted questionnaire [17, 18] before the clinical assessment.

### Statistical analysis

Data were analyzed separately for European children and those with an immigrant background. Caries experience was first analyzed through cross-tabulations and adjusted for age and sex through regression analysis. Both the questionnaire and oral examination forms were manually checked for the completion of the required information.

An algorithm about the household income was developed derived by the modified Organization for Economic Co-operation and Development (OECD) equivalence scale (https://www.oecd.org) [19]. The household was assembled via allocating scores to each person in a household and then summing up the equivalence points of all household members [14]. The factor was then categorized in 4 levels, with quartile 1 being the lowest and quartile 4 being the highest. The five Italian areas were ordered following the mean Gross National Product (GNP) *per capita* starting from the highest (North-West, North-East, Central, South, Islands). As proxy measurements of family’s socio-economic status, the occupational profile and the educational level of both parents and the number of offspring were recorded. The parents’ occupational profile was categorized into 3 levels: un-employment/unskilled jobs as the lowest, skilled jobs/qualified jobs and white-collar jobs as the highest. The educational level was categorized in Low (no education/primary education/lower secondary education), Intermediate (upper secondary) and High (degree, master or doctorate) [20]. Oral health habits (frequency of toothbrushing and fluoride supplements beyond toothpaste), diet (duration of breast-feeding, use of sweetened pacifier at night) and life-style behaviors (parents/guardians smoking habits) were also collected. For each explanatory variable, the group with the most favorable situation was chosen as a reference point from which to measure disparities. The regression between the caries experience (CariesEx) in each household quartile was computed and the Slope Index of Inequality (SII) [21], as of the mid-point value of CariesEx score in each household group, was calculated.

As a proxy for health inequality, a social gradient was generated as the weighted sum of the worst circumstances deriving from social explanatory variables. Children were stratified into four social gradient levels based on their number of worst options: ‘‘best,’’ with up to two; ‘‘good,’’ with three; ‘‘bad,’’ with four; and ‘‘worst,’’ with five or more. Multivariate regression models were used to elucidate the associations between all explanatory variables and health outcome (namely the caries prevalence). Assuming an overdispersion and excess of zero in total sample and in children with European background, the Zero-Inflated Negative Binomial logistic regression was used; while, a logistic regression was used in children with non-European background, assuming a high caries prevalence. Different additional analyses were performed and Mantel Haenszel trends of odds adjusted by immigrant background and area of living were calculated to study the existence, dimensions and direction of the social gradients in oral health.

Stata/SE 16.1 for Mac (Intel 64-bit) and SPPS 27 were used to account for the complex data structure.

## Results

**Fig. 1:**
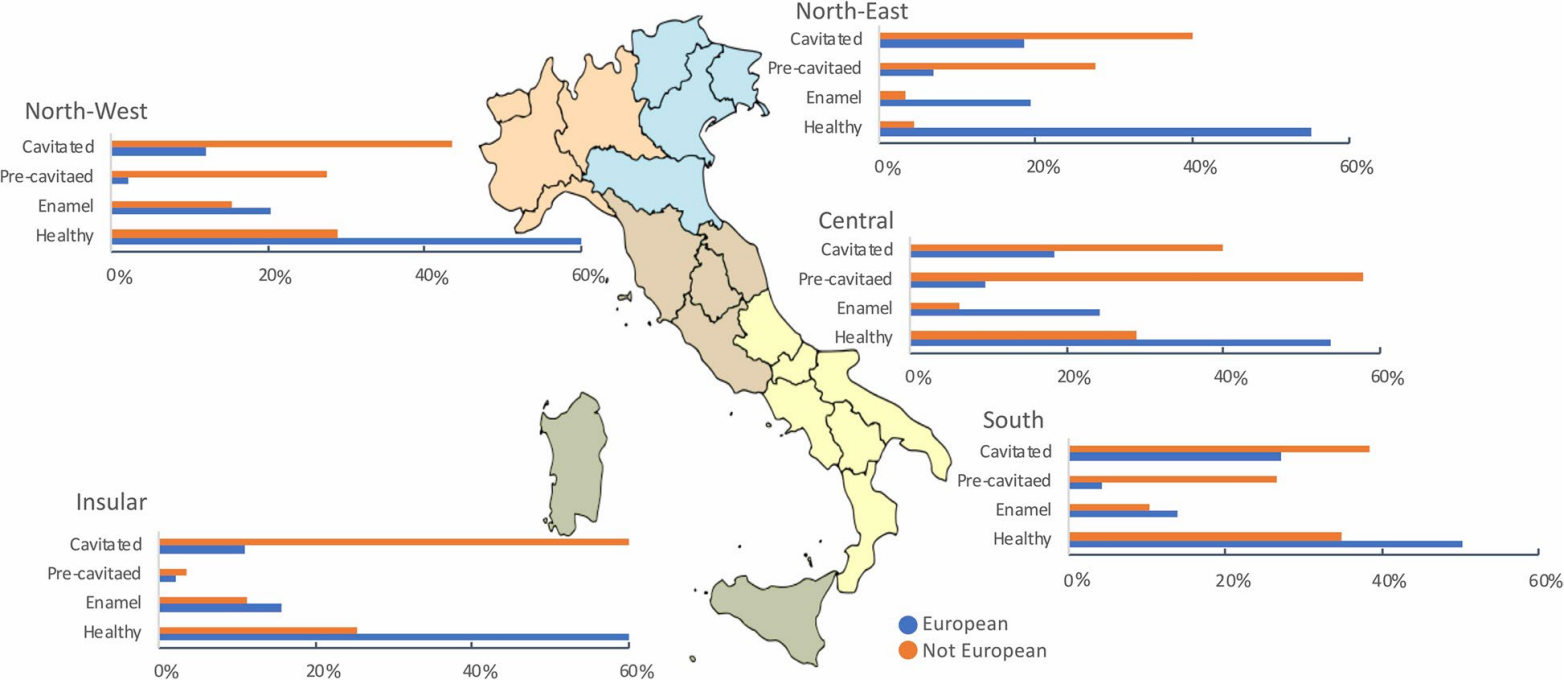
Caries severity, expressed as caries free, enamel, pre-cavitated and cavitated lesions across the different Italian areas by European and non-European immigrant

Overall, 6,825 children (52.8% females) were examined, of which 883 (12.9%) were of non-European background. The distribution of caries figures for both European and non-European children across the five different Italian areas (Fig. 1) showed that caries prevalence was statistically significant higher in children with non-European background compared to European children (72.6% *vs* 41.6% respectively χ^2^ = 297.29 *p* < 0.01).

A not equal distribution by sex across both European and non-European groups was recorded; a higher caries prevalence in females in the non-European group (80.8%) was found, while in European background sample a higher prevalence in males (61.1%) was observed (*data not in table*).

Descriptive and bivariate analysis (by European and non-European background) is displayed in Table 1.

**Table 1:** Caries experience in European and non-European children across geographical areas, household, parents’ occupation and educational level

		**European**			**Non-European background**		
		**Caries-free**	**Caries-Ex**	**OR (95%CI)**	**Caries-free**	**Caries-Ex**	**OR (95%CI)**
		**n (%)**	**n (%)**		**n (%)**	**n (%)**	
**Geographical areas**^a^	North-West	1037 (65.2)	554 (34.8)	reference	88 (28.9)	217 (71.1)	reference
	North-East	662 (55.3)	536 (44.7)	1.52 (1.29–1.77)	4 (4.3)	88 (95.7)	8.92 (3.07–25.90)
	Central	614 (53.7)	530 (46.3)	1.62 (1.38–1.89)	42 (29.0)	103 (71.0)	0.99 (0.64–1.54)
	South	685 (50.1)	681 (49.9)	1.86 (1.60–2.16)	79 (35.0)	147 (65.0)	0.75 (0.52–1.09)
	Islands	471 (73.2)	172 (26.8)	0.68 (0.55–0.84)	29 (25.2)	86 (74.8)	1.20 (0.74–1.96)
		*Mantel Haenszel trend of odds* χ^*2*^ = *3.94 p* = *0.03*			*Mantel Haenszel trend of odds* χ^*2*^ = *1.55 p* = *0.22*		
**Household**	1^st^ quartiles	511 (42.1)	702 (57.9)	8.68 (6.66–11-31)	106 (24.7)	324 (75.3)	22.41 (8.50–59.14)
	2^nd^ quartiles	646 (36.9)	1107 (63.1)	10.83 (8.35–14.03)	92 (22.8)	311 (77.2)	24.79 (9.22–66.63)
	3^rd^ quartiles	1674 (74.8)	563 (25.2)	2.12 (1.68–2.68)	44 (88.0)	6 (12.0)	reference
	4^th^ quartiles	638 (86.3)	101 (13.7)	reference	–	–	–
		*Mantel Haenszel trend of odds* χ^*2*^ = *735.80 p* < *0.01*			*Mantel Haenszel trend of odds* χ^*2*^ = *28.18 p* < *0.01*		
**Parents’ occupation**	Un-employment/unskilled	559 (48.3)	598 (51.7)	3.28 (2.83–3.79)	235 (29.7)	556 (70.3)	0.16 (0.04–0.67)
	Skilled jobs/qualified	429 (39.9)	645 (60.1)	4.60 (3.94–5.37)	5 (8.3)	55 (91.7)	0.73 (01.13–4.05)
	White-collar	2319 (75.4)	758 (24.6)	reference	2 (6.2)	30 (93.8)	reference
		*Mantel Haenszel trend of odds* χ^*2*^ = *382.30 p* < *0.01*			*Mantel Haenszel trend of odds* χ^*2*^ = *18.37 p* < *0.01*		
**Parents’ education**	Low	187 (41.9)	259 (58.1)	2.12 (1.73–2.59)	209 (28.3)	529 (71.7)	1.04 (0.64–1.67)
	Intermediate	1181 (58.4)	841 (41.6)	1.09 (0.97–1.22)	6 (11.5)	46 (88.5)	3.13 (1.17–8.40)
	High	2101 (60.5)	841 (41.5)	reference	27 (29.0)	66 (71.0)	reference
		*Mantel Haenszel trend of odds* χ^*2*^ = *37.01 p* < *0.01*			*Mantel Haenszel trend of odds* χ^*2*^ = *0.38 p* = *0.54*		

*OR* Odds Ratio,  *95%CI* 95% Confidence Interval. The Mantel Haenszel trend of odds was calculated.

^a^Geographical areas were ordered following the mean Gross National Product (GNP) per capita for each Italian area

The caries prevalence was statistically associated (Mantel Haenszel trend of odds χ^2^ = 3.94 *p* = 0.03) to the area of living ordered by GNP among European children: the caries prevalence increases as the GNP decreases. In children with a non-European background, caries prevalence was not associated with the area of living. The caries prevalence (CariesEx) was statistically significant different across the different household income quartiles, the parents’ occupation both in children with European and non-European background (*p* < 0.01). Caries figure was also statistically significant associated (*p* < 0.01) to behavioral factors: Breastfeeding, Pacifier at night, toothbrushing frequency, Cariogenic diet, Smoking habit of parents (see Additional file 1).

**Fig. 2:**
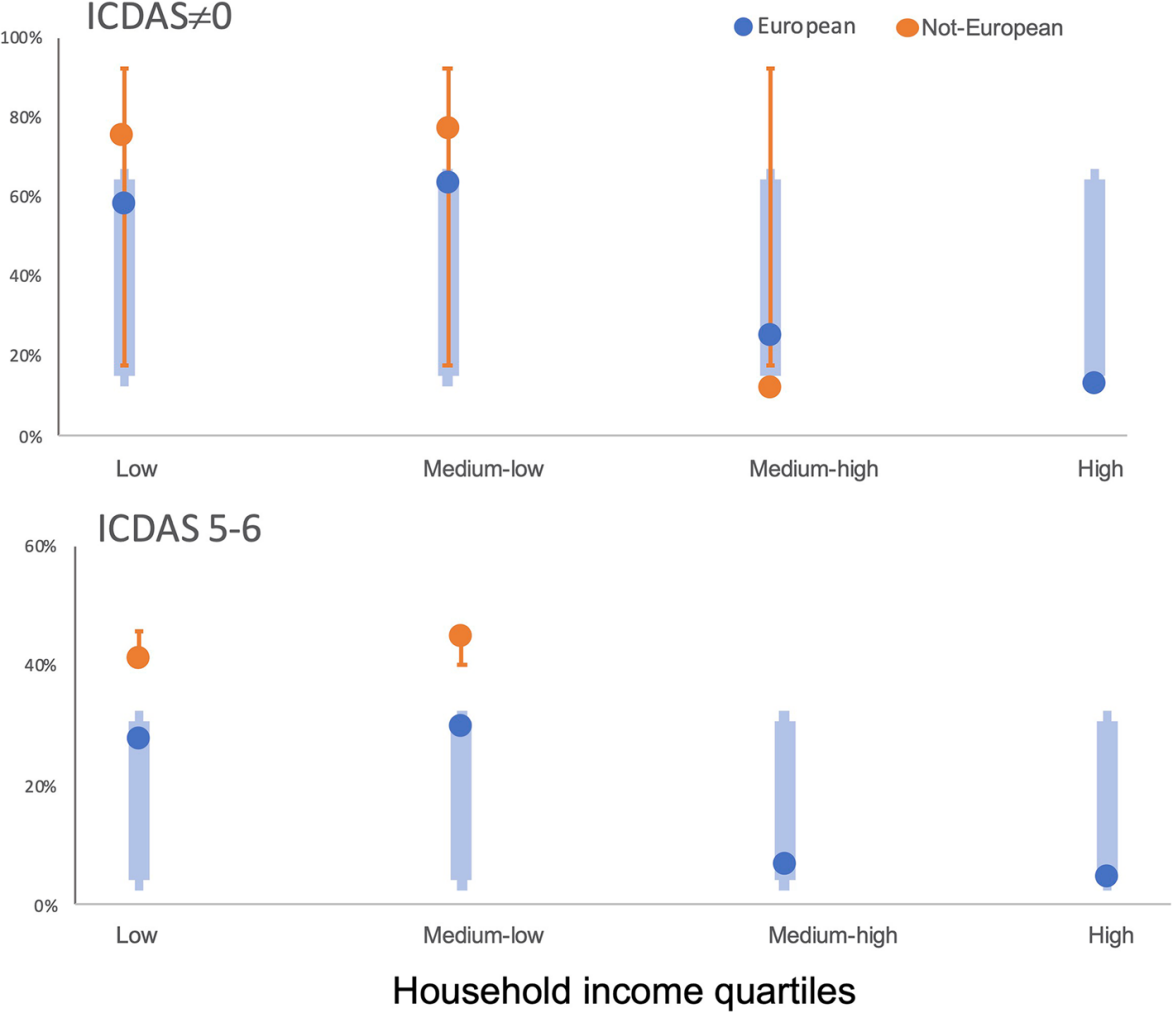
Outcomes by quartiles of income regarding caries experience (ICDAS ≠ 0) and cavitated lesions (ICDAS 5–6) among children with European and non-European background

The social levels, by equivalized household income, for caries experience and cavitated lesions prevalence for both European and non-European children are clearly demonstrated in Fig. 2: children in the highest household income group had lower levels of caries (either caries experience and cavitated lesions prevalence) than their counterparts in the lowest income group.

The coefficients from the multivariate analysis with negative values indicate that the social disparity in caries favors the more socially advantaged children (Table 2).

**Table 2:**
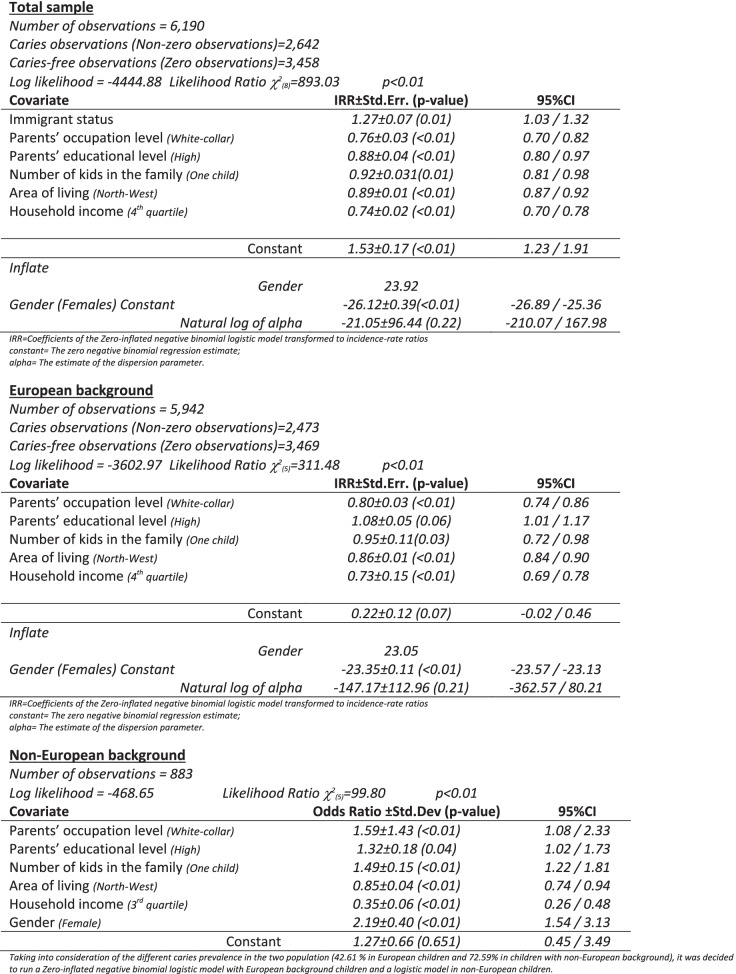
Multivariate regression coefficients of caries prevalence in total sample and children with European and Immigrant background

In total sample, all the estimated coefficients namely the Immigrant status, the highest occupational and educational level of the parents, only one kid in the family, living in the North-Western Italian area and a high household income, of the Zero-inflated negative binomial logistic model (transformed to incidence rate ratios), were statistically significant associated (*p* = 0.01 or *p* < 0.01) to caries prevalence. In European children, the estimated coefficients (Parents’ occupation level, Number of kids in the family, Area of living and Household income) of the Zero-inflated negative binomial logistic model transformed to rate ratios, were statistically significant associated (*p* < 0.01) to caries prevalence. White-collar parents, family living in North-West and highest household income (4^th^ quartile) played a protective effect on caries prevalence. For the non-European children, all the social background factors were statistically significant associated to caries prevalence (*p* < 0.01, except for Parents’ educational level, *p* = 0.04) with an increment of risk in children with white-collar parents and parents with a high education level, while a protective effect was observed for highest quartile of household (3^rd^ quartile) and highest GDP-related area (North-West area). All behavioral and life-style habits considered in the survey were statically significant associated (*p* < 0.01) to caries prevalence in total sample and in children with European background, while in non-European children the use of pacifier at night was not statistically significant associated to caries prevalence (see Additional file 2).

Overall, the social gradient is clearly able to demonstrate how the worst social factors expose children to a greater risk for each caries level (except cavitated) and the overall caries prevalence (*data not in table)*. The social gradient was statistically significant associated (*p* < 0.01) to the different caries levels and experience in children with European background (Table 3).

**Table 3:** Association between social gradient (from best to worst) and caries levels (enamel lesions, pre-cavitated lesions, cavitated lesions) and total caries prevalence adjusted by areas of living

**European background**				
**Social Gradient**	**Enamel Lesions *(ICDAS 1/2)***	**Pre-cavitated Lesions *(ICDAS 3/4)***	**Cavitated Lesions *(ICDAS 5/6)***	**Overall Caries prevalence *(ICDAS ≠ 0)***
	*OR (95%CI)*	*OR (95%CI)*	*OR (95%CI)*	*OR (*_*95%*_*CI)*
**Best**	*reference*	*reference*	*reference*	*reference*
**Good**	1.34 (1.17–1.54)	3.22 (2.35–4.40)	4.30 (3.61–5.13)	3.24 (2.88–3.66)
**Bad**	1.31 (1.04–1.64)	5.81 (3.74–9.03)	2.87 (2.27–3.64)	2.78 (2.31–3.34)
**Worst**	3.97 (1.14–13.83)	6.61 (0.90–48.60)	6.42 (1.99–20.75)	16.77 (3.56–83.74)
*Mantel Haenszel trend of odds*	χ^*2*^ = *19.02 p* < *0.01*	χ^*2*^ = *60.28 p* < *0.01*	χ^*2*^ = *203.23 p* < *0.01*	χ^*2*^ = *325.97 p* < *0.01*
**Non-European background**				
**Social Gradient**	**Enamel Lesions *(ICDAS 1/2)***	**Pre-cavitated Lesions *(ICDAS 3/4)***	**Cavitated Lesions *(ICDAS 5/6)***	**Overall Caries prevalence *(ICDAS ≠ 0)***
	*OR (95%CI)*	*OR (95%CI)*	*OR (95%CI)*	*OR (*_*95%*_*CI)*
**Best**	*reference*	*reference*	*reference*	*reference*
**Good**	–	–	3.62 (0.10–124.40)	19.87 (0.04- > 1000)
**Bad**	–	–	0.23 (0.01–7.43)	–
**Worst**	–	–	0.52 (0.11–2.35)	7.53 (0.33–170.47)
*Mantel Haenszel trend of odds*	––	––	χ^*2*^ = *0.37 p* = *0.54*	χ^*2*^ = *23.32 p* < *0.01*

*OR* Odds Ratio,  *95%CI* 95% Confidence Interval and the Mantel Haenszel trend of odds were calculated

For enamel lesions and pre-cavitated lesions very few observations were present so the association between Social Gradient was not run

Subjects with the worst gradient had usually the highest Odds-ratios for caries prevalence. As caries experience was so high in children with non-European background, no social gradient was detected.

## Discussion

Caries, especially in disadvantaged groups, remains a huge public health problem around the world with an increasing burden of untreated caries [21, 22]. In European Union, an overall increment in caries prevalence was reported in young children with lower socioeconomic status and/or an immigrant background as they are the most vulnerable group within countries [23]. The present survey confirmed it, describing a high caries prevalence in young children living in Italy, especially in those with a non-European background. The association between socioeconomic inequality and caries disease is more pronounced in children with a European background than in children with a non-European background due to the different caries figures in the two groups.

The main finding of this survey suggests that socioeconomic inequality is reflected in a higher caries prevalence and experience. Caries severity fluctuates in a statistically significant way in the different geographical areas. Children with an immigrant background showed a higher prevalence of cavitated lesions, especially in the Insular area of Italy where a low GNP is present.

In this survey, a social gradient in caries distribution was evident among Italian children with a European background, highlighting how income and social status are important determinants in caries figures. The social gradient was less evident in children with a non-European background for different possible reasons. First of all, a not uniform distribution of not European background children in the different social quartiles was noted, since a very poor number of children belonged to families in the Medium–high and non-one in the Highest quartile. This situation is not surprising since contrary to what is observed for Italian natives, immigrants does not contribute to getting access to high-paying occupations [24]. Another reason might be related to the very high caries prevalence in this group which also renders the gradient irrelevant. A high caries prevalence in immigrant population might be due to multiple factors such as sociodemographic, socioeconomic, or cultural factors, probably mediated by specific behaviors such as poor oral hygiene habits and high consumption of foods and drink high in sugars [25]. It is hard to fully explicate health differences because no single factor can generally account for them [26], due to the combined and cumulative effect of risk factors and under different living conditions such as migrant status in this survey. Typically, there is a gradual, if not a linear, trend of declining health status confined to a social group that is at the extreme end of the social scale, while all other groups have relatively good levels of health [1].

In Italy, the fraction of Gross National Product spent on healthcare has been significant reduced during the last two decades as described above, and oral health is lagging behind. Oral care is mainly almost exclusive provided by private practitioners and it is mainly financed by direct payment of the patient or, to a lesser extent, through private insurance schemes. It is consequent that family income is a determining factor in the access of care. Data of the present survey show how caries distribution is associated with mean GNP of the living area of the family.

Among the social variables considered, the highest parents’ educational level plays an ambivalent role: it plays as a protective factor for the total sample, while it increases the caries risk when European background and non-European children are individually analyzed. The increased risk of caries found in non-European children could be related to a higher income that, if not associated to adequate knowledge and behavior, might expose children to a greater risk of disease. However, it is necessary to underline that the number of parents in this group with the highest level of employment was rather small, as proof of what has already been reported above.

Belonging to a family with one play a protective role in children with a European background, while it plays a role in favoring caries in the group of children with a non-European background. Women with a European background in Italy have fewer children (1.19 per capita) than those with a non-European background (1.98 per capita) [27]. In addition, women with a European background have children at a later age, because they are engaged longer in studies and then in looking for a job. This situation leads families with a European background to have fewer children, to have them at an age where there is greater awareness of healthy habits and when there is greater economic well-being. All these factors contribute to reducing the risk of caries. Conversely, women with non-European background have more children at an earlier age than European ones, and these conditions often lead them to a lower educational level and to a lower income or no income at all, conditions favoring a higher risk of caries in offspring [28].

Even if caries is not considered a sex-related disease, many caries risk factors provide a sex bias, placing females at a higher risk, as well as differences in access to dental care justify a higher rate of untreated caries. [29, 30]. In the present survey, this different caries figures between females and males were only found in children with a non-European background.

In the previous pathfinder on pre-school children [14], subjects with a non-European background were not considered since the percentage of immigrants in Italy was low at that time. Despite a different epidemiologic index used (dmfs), a non-homogeneous geographical distribution of caries was already evident, which has not changed to date.

At the best of authors’ knowledge, the present survey is the first National pathfinder carried-out in primary dentition using ICDAS as epidemiological caries index. Another strength of the present survey is the high number of subjects screened, which makes the data representative of the preschool population of each geographic area of the Country. A weakness is the lack of individual income data, since only macroeconomic data of the geographical area of living were available and used for the analyses.

## Conclusions

The data derived from the present survey show:

- In Italy, caries disease in preschool children is an unsolved public health problem, especially in areas where income is low and among the most socially and economically unprivileged groups.
- The current caries figure requires a political effort and investments in caries promotion and prevention programs in the very early stages of children’s life, especially addressed to family with a non-European background and those living in areas with the lowest economic status.
- The generated social gradient showed a high association with caries disease in children with a European background, while a less extent of it was noted in children with non-European background.

## Supplementary Information

**Additional file 1: Table 1S**. Caries prevalence in children divided by European and Non-European background across Breastfeeding, Pacifier at night, Brushing frequency, Cariogenic diet, Smoking habit.

**Additional file 2: Table 2S. **Multivariate regression coefficients of caries prevalence in total sample and children with European and Immigrant background (behavioral habits).

**Additional file 3: Supplementary S3.** Row data.

## Data Availability

All data generated or analysed during this study are included in this published article and its supplementary information files [dataraw.xlsx].
