# Supplementary material for: Inequalities in caries among pre-school Italian children with different background
Source: BMC Pediatr. 2022 Jul 23;22:443. doi: 10.1186/s12887-022-03470-4 (PMC9308358; doi:10.1186/s12887-022-03470-4)
Supplement: Supplementary file 1 — Additional file 1: Table 1S. Caries prevalence in children divided by European and Non-European background across Breastfeeding, Pacifier at night, Brushing frequency, Cariogenic diet, Smoking habit. [file 12887_2022_3470_MOESM1_ESM.docx]

**Table 1S**. Caries prevalence in children divided by European and Non-European background across Breastfeeding, Pacifier at night, Brushing frequency, Cariogenic diet, Smoking habit.

|  |  | **European** | | | | | **Non-European** | | | | | | |
| --- | --- | --- | --- | --- | --- | --- | --- | --- | --- | --- | --- | --- | --- |
| **Breastfeeding** |  | **Caries-free** | **Caries-Ex** | | **OR (_95%_CI)** | | **Caries-free** | | **Caries-Ex** | | **OR (_95%_CI)** | | |
|  |  | **n (%)** | **n (%)** | |  | | **n (%)** | | **n (%)** | |  | | |
|  | No | 1569 (67.45) | 757 32.55) | | 1.69 (1.42-2.02) | | 7 (3.38) | | 200 (96.62) | | 17.57 (7.46-41.37) | | |
|  | ≤6 months | 666 (69.30) | 295 (30.70) | | 1.56 (1.27-1.91) | | 1 (2.04) | | 48 (97.96) | | 29.52 (3.74-94.98) | | |
|  | >6-≤12 months | 763 (77.86) | 217 (22.14) | | reference | | 123 (38.08) | | 200 (61.92) | | reference | | |
|  | >12-≤18 months | 298 (23.69) | 960 (76.31) | | 11.32 (8.94-14.34) | | 92 (48.94) | | 96 (51.06) | | 0.641 (0.44-0.92) | | |
|  | >18 months | 173 (41.49) | 244 (58.51) | | 4.96 (3.81-6.45) | | 19 (16.38) | | 97 (83.62) | | 3.14 (1.80-5.45) | | |
|  |  | *Mantel Haenzel trend of odds χ^2^=451.98 p<0.01* | | | | | *Mantel Haenzel trend of odds χ^2^=52.85 p<0.01* | | | | | | |
| **Pacifier at night** | No | 2959 (71.13) | 1201 (28.87) | | reference | | 241 (43.35) | | 315 (56.65) | | reference | | |
|  | Yes | 510 (28.62) | 1272 (71.38) | | 6.14 (5.37-7.02) | | 1 (0.31) | | 326 (99.69) | | 49.42 (26.88-2.31^e+03^) | | |
|  |  | *Mantel Haenzel trend of odds χ^2^=927.73 p<0.01* | | | | | *Mantel Haenzel trend of odds χ^2^=191.49 p<0.01* | | | | | | |
| **Brushing frequency** | Once a day | 0 (0.00) | 71 (100.00) | | -- | | | 0 (0.00) | 118 (100.00) | | -- | | |
|  | Twice a day | 199 (14.33) | 1109 (85.67) | | 16.13 (13.34-19.51) | | | 0 (0.00 | 157 (100.00) | | -- | | |
|  | More than twice a day | 3270 (72.96) | 1212 (27.04) | | reference | | 242 (39.80) | | 366 (60.20) | | reference | | |
|  |  | *Mantel Haenzel trend of odds χ^2^=1553.78 p<0.01* | | | | | *Mantel Haenzel trend of odds χ^2^= 128.27 p<0.01* | | | | | | |
| **Cariogenic diet** | No | 2449 (81.07) | | 572 (18.93) | | reference | | 102 (53.68) | | 88 (46.32) | | | reference |
|  | Yes | 1020 (34.92) | | 1901 (65.08) | | 7.98 (6.98-9.12) | | 140 (20.20) | | 553 (79.80) | | | 4.57 (3.20-6.55) |
|  |  | *Mantel Haenzel trend of odds χ^2^=1301.33 p<0.01* | | | | | *Mantel Haenzel trend of odds χ^2^=83.93 p<0.01* | | | | | | |
| **Smoking habit** | No | 2813 (76.44) | | 867 (23.56) | | reference | | 235 (43.84) | | 301 (56.16) | | reference | |
|  | Yes | 656 (29.00) | | 1606 (71.00) | | 7.94 (6.95-9.08) | | 7 (2.02) | | 340 (97.98) | | 37.92 (15.98-89.97) | |
|  |  | *Mantel Haenzel trend of odds χ^2^=1297.32 p<0.01* | | | | | *Mantel Haenzel trend of odds χ^2^=185.00 p<0.01* | | | | | | |

*Odds Ratio (OR) and 95% Confidence Interval (_95%_CI). The Mantel Haenszel trend of odds was calculated.*
