# Supplementary material for: Inequalities in caries among pre-school Italian children with different background
Source: BMC Pediatr. 2022 Jul 23;22:443. doi: 10.1186/s12887-022-03470-4 (PMC9308358; doi:10.1186/s12887-022-03470-4)
Supplement: Supplementary file 2 — Additional file 2: Table 2S. Multivariate regression coefficients of caries prevalence in total sample and children with European and Immigrant background (behavioral habits). [file 12887_2022_3470_MOESM2_ESM.docx]

**Table 2S.** Multivariate regression coefficients of caries prevalence in total sample and children with European and Immigrant background (behavioral habits)

**Total sample**

*Number of observations = 6,190*

*Caries observations (Non-zero observations)=2,642*

*Caries-free observations (Zero observations)=3,458*

*Log likelihood = -4444.88 Likelihood Ratio χ^2^_(8)_=893.03 p<0.01*

| **Covariate** | **IRR±Std.Err. (p-value)** | **_95%_CI** |
| --- | --- | --- |
| Immigrant status | *1.31±0.06 (<0.01)* | *1.20 / 1.44* |
| Breastfeeding *(>6≤12 months)* | *1.10±0.014 (<0.01)* | *1.07 / 1.13* |
| Pacifier at night *(Yes)* | *1.27±0.06 (<0.01)* | *1.16 / 140* |
| Brushing frequency *(> twice a day)* | *0.77±0.03 (0.01)* | *0.72 / 0.83* |
| Cariogenic diet *(No)* | *2.02±0.10 (<0.01)* | *1.83 / 2.23* |
| Smoking habit *(No)* | *1.80±0.07 (<0.01)* | *1.66 / 1.94* |
| constant | *0.24±0.02 (<0.01* | *0.20 / 0.28* |
| *Inflate*  *Gender* | *17.48* |  |
| *Gender (Females) Constant* | *-37.41±323.56 (1.00) -* | *-63.42 / 643.17* |
| *Natural log of alpha* | *-181.15±71.98 (0.02)* | *-322.24 / -40.06* |

*IRR=Coefficients of the Zero-inflated negative binomial logistic model transformed to incidence-rate ratios*

*constant= The zero negative binomial regression estimate;*

*alpha= The estimate of the dispersion parameter.*

**European background**

*Number of observations = 5,942*

*Caries observations (Non-zero observations)=2,473*

*Caries-free observations (Zero observations)=3,469*

*Log likelihood = -3932.83 Likelihood Ratio χ^2^_(5)_=1032.19 p<0.01*

| **Covariate** | **IRR±Std.Err. (p-value)** | **_95%_CI** |
| --- | --- | --- |
| Breastfeeding *(>6≤12 months)* | *1.16±0.02 (<0.01)* | *1.12 / 1.19* |
| Pacifier at night *(Yes)* | *1.21±0.06 (<0.01)* | *0.09 / 1.34* |
| Brushing frequency *(> twice a day)* | *0.67±0.03 (<0.01)* | *0.61 / 0.73* |
| Cariogenic diet *(No)* | *2.05±0.11 (<0.01)* | *1.84 / 2.29* |
| Smoking habit *(No)* | *1.81±0.08 (<0.01)* | *1.65 / 1.98* |
| constant | *0.27±0.03 (<0.01)* | *0.22 / 0.33* |
| *Inflate*  *Sex* | *19.00±6.68 (1.00)* | *-1.34 / 1.34* |
| Sex (Female) Constant | *-38.75±6.69 (1.00)* | *-1.34 / 1.34* |
| *Natural log of alpha* | *-24.84±81.03 (0.76)* | *-183.64 / 133.97* |

*IRR=Coefficients of the Zero-inflated negative binomial logistic model transformed to incidence-rate ratios*

*constant= The zero negative binomial regression estimate;*

*alpha= The estimate of the dispersion parameter.*

**Non-European background**

*Number of observations = 883*

*Log likelihood = -468.65 Likelihood Ratio χ^2^_(5)_=99.80 p<0.01*

| **Covariate** | **Odds Ratio ±Std.Dev (p-value)** | **_95%_CI** |
| --- | --- | --- |
| Breastfeeding *(>6≤12 months)* | *0.32±0.04 (<0.01)* | *0.24 / 0.43* |
| Pacifier at night *(Yes)* | *2.40±2.75 (0.44)* | *0.25 / 22.64* |
| Brushing frequency *(> twice a day)* | *omitted* | *----* |
| Cariogenic diet *(No)* | *0.60±0.16 (0.06)* | *0.35 /1.02* |
| Smoking habit *(No)* | *0.15±0.06 (<0.01)* | *0.07 / 0.92* |
| Constant | *9.67±4.21 (<0.01)* | *4.11 / 22.72* |

*Taking into consideration of the different caries prevalence in the two population (42.61 % in European children and 72.59% in children with non-European background), it was decided to run a Zero-inflated negative binomial logistic model with European background children and a logistic model in non-European children.*
